# Oral functional impairment may cause malnutrition following oral cancer treatment in a single-center cross-sectional study

**DOI:** 10.1038/s41598-022-19177-6

**Published:** 2022-08-30

**Authors:** Reon Morioka, Yuhei Matsuda, Akira Kato, Tatsuo Okui, Satoe Okuma, Hiroto Tatsumi, Takahiro Kanno

**Affiliations:** Department of Oral and Maxillofacial Surgery, Shimane University Faculty of Medicine, 89-1 Enya-cho, Izumo, Shimane 693-8501 Japan

**Keywords:** Head and neck cancer, Oral cancer, Dentistry

## Abstract

Oral dysfunction and dysphagia after oral cancer treatment are linked to altered nutritional status. We aimed to identify specific oral functions related to nutritional status. We conducted a cross-sectional study from September 2019 to December 2021, recruited 75 participants (median age: 72.0 years), including 52 males and 23 females, collected background data, and evaluated oral function. The Mini Nutritional Assessment-Short Form (MNA-SF) scores were divided into three groups (normal nutritional status, at risk of malnutrition, and malnourished), and a multi-group comparison was conducted for each oral function measurement (microorganisms, oral dryness, occlusal force, tongue pressure, masticatory function, and Eating Assessment Tool [EAT-10]). The primary tumor site was the tongue in 31 patients (41.3%), gingiva in 30 (40.0%), and others in 14 (18.7%). Multiple comparisons revealed significant differences in occlusal force, tongue pressure, masticatory function, and EAT-10 levels, categorized as Type I (Transport type) and Type III (Occlusion type) postoperative oral dysfunctions, between each MNA-SF group. Multiple regression analysis showed a statistically significant association with MNA-SF in terms of masticatory function and EAT-10 levels, categorized as Type I. Type I and Type III are risk factors for malnutrition, confirming that different types of postoperative oral dysfunction require unique nutritional guidance.

## Introduction

The disease-specific survival rates of oral cancer patients have increased with the development of surgical techniques, molecular targeted drugs, and immune checkpoint inhibitors, as well as the application of radiotherapy, represented by intensity-modulated radiation therapy^1^. With an increase in the number of oral cancer patients due to the increased survival rate after treatment and the aging of the population, the number of oral cancer patients living with the sequelae of oral cancer treatment is increasing^2^. According to the current National Comprehensive Cancer Network (NCCN) guidelines, resection is often the first-line treatment for oral cancer^3^. Thus, there is a wide range of sequelae after oral cancer treatment, such as xerostomia, dysphagia, speech disorder, tooth loss, chronic pain, body image concerns, anxiety/depression, trismus, rampant tooth decay, osteonecrosis, and malnutrition; it was also reported that the quality of life of patients with oral cancer deteriorated after treatment^4,5^. In particular, restriction of food intake is a sequela that can lead to malnutrition. Approximately one-quarter of patients who have undergone oral cancer treatment experience a decrease in food intake^6,7^.

**Figure 1:**
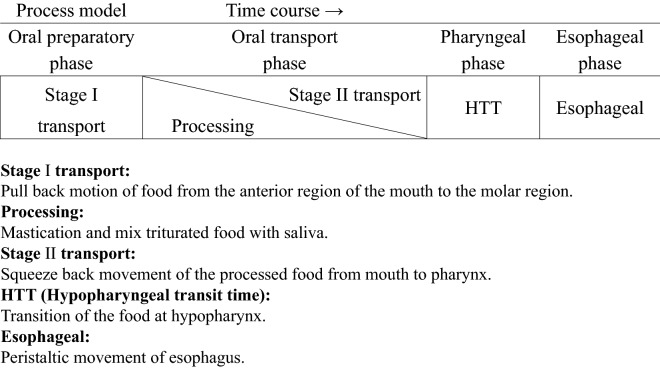
Swallowing process model.

The swallowing process is remarkably complex, involving six cranial nerves, multiple muscle groups, and cortical and subcortical brain signals that must be precisely coordinated within a few seconds^8^. In recent years, swallowing behavior has often been described using process models (Fig. 1)^9^. A four-phase continuous model of swallowing and process models are used to understand dysphagia caused by dementia, stroke, amyotrophic lateral sclerosis, and oral cancer^10^. In oral cancer treatment, sudden anatomical structural displacement and lack of motor sequence lead to postoperative impairment of oral function, which impairs Stage I transport, processing, and Stage II transport. Hence, there is a need for more suitable methods to evaluate postoperative dysphagia in oral cancer patients. The Matsuda–Kanno classification of postoperative oral dysfunction is useful for comprehending different types of swallowing dysfunction, as previously reported^11^. Briefly, three components were identified using principal component analysis in the Matsuda–Kanno classification^11^. Type I (Transport type) includes masticatory function, Eating Assessment Tool (EAT-10), and tongue pressure. It is widely considered a conceptual disorder that affects both Stage I and Stage II transport. Type II (Oral hygiene type) consists of items related to bacterial counts, oral health perception, and oral dryness.

Oral dryness has been reported to be an accelerator for an increased number of oral microorganisms due to the decreased rate of salivary flow^12,13^. A decreased rate of salivary flow has been reported to be a risk factor for periodontal disease and dental caries^14^. Tooth loss due to these common dental diseases can lead to decreased masticatory and swallowing function, possibly leading to malnutrition^15,16^. Type III (Occlusal type) consists of occlusal force alone. This component is thought to be an independent component because its elements are very different from those of other components^11^. Therefore, recent studies have clarified the details of postoperative oral function following evidence-based oral cancer treatment guided by the NCCN guidelines^3^.

However, there are still some problems with nutritional instructions for oral cancer patients. Although there are reports that early intervention by a dietitian may improve the nutritional status of patients with oral cancer, two randomized controlled trials could not confirm the effectiveness of this intervention^17,18^. Therefore, we hypothesized that different types of postoperative oral dysfunction require different nutritional guidance and conducted a study to clarify the relationship between various postoperative oral functions and nutritional status. It is assumed (null hypothesis H0) that Type I–III oral dysfunctions are not associated with nutritional status.

## Methods

### Data collection

Participants in this cross-sectional study were enrolled according to the following criteria: (1) patients diagnosed and treated for oral squamous cell carcinoma based on NCCN guidelines by a single surgical team in a single center; (2) patients who visited the Department of Oral and Maxillofacial Surgery/Oral Care Center at Shimane University Hospital (Shimane, Japan); (3) patients aged 20 years or older; and (4) patients who could understand the questions and answer the questionnaire. Participants were excluded according to the following criteria: (1) cases of recurrent or metastatic oral cancer and (2) cases of drop-out due to death from perioperative complications^3^. All data were collected just before the patient was discharged from the hospital. All treatments were performed at the hospital, and the time of discharge was defined as the point at which the attending physicians judged, based on the treatment workup and course according to the Japanese medical insurance system after definitive oral cancer treatment guided by the NCCN guideline, that there were no systemic or local complications and that the patient could reintegrate into social activities and life. The study period lasted from September 2019 to December 2021, and data were collected using a sequential sampling method. This study was approved by the Institutional Review Board of the Ethics Committee of the Shimane University Faculty of Medicine (approval number: 4041) on September 30, 2019. Also, all methods were performed in accordance with Declarations of Helsinki. Written informed consent was obtained from each participant prior to participation in the study.

### Background data

We sampled the following variables as background data: sex, age (years), body mass index (BMI, kg/m^2^), alcohol consumption (regular drinker or not), Brinkman index, Eastern Cooperative Oncology Group performance status, primary tumor site, cancer stage based on the criteria of the Union for International Cancer Control (version 8), treatment methods (surgery/surgery and adjuvant radiotherapy/surgery and adjuvant chemoradiotherapy), presence of neck dissection, presence of reconstructive surgery, and number of teeth.

### Oral function measurement

The method recommended by the Japanese Society of Gerodontology in its position paper was adopted to measure oral function^19^. However, tongue-lip motor function could not be assessed in patients with tongue defects caused by oral cancer treatment; therefore, it was removed from the assessment items.

#### Microorganisms

The number of microorganisms was measured by collecting samples from the center of the tongue dorsum using a rapid oral detection apparatus (Bacterial counter; Panasonic Healthcare, Tokyo, Japan). The number of microorganisms indicated by the bacterial counter and the grade were recorded.

#### Oral dryness

Oral dryness was measured using an oral moisture checker (Mucus; Life, Saitama, Japan), and the median of three measurements on the dorsum of the tongue was taken as the data. Measurements were taken on the healthy side if there was a reconstructed flap due to a defect in the tongue, and in the middle of the flap if a total resection had been performed.

#### Occlusal force

The occlusal force was measured using a pressure-sensitive paper (Dental Prescale Occluzer; GC, Tokyo, Japan) by clenching for 3 s at the intercuspal position. If the subject had a denture, the occlusal force was measured with the denture in place.

#### Tongue pressure

Tongue pressure was measured at the center of the dorsum of the tongue using a tongue pressure measuring instrument (TPM-01; JMS, Hiroshima, Japan).

#### Masticatory function

Masticatory function was measured using a masticatory ability testing system (Gluco Sensor GS-II; GC, Tokyo, Japan).

#### EAT-10

Swallowing function was assessed using a 10-question questionnaire measured on a 5-point Likert scale (0 = no problem; 4 = severe problem) developed by Belafsky in 2008^20^. The EAT-10 has a maximum total score of 40, with higher scores indicating poor swallowing function.

### Swallowing function measurement

#### Functional oral intake scale

The functional oral intake scale (FOIS) has a maximum total score of 40, with higher scores indicating poor swallowing function^21^.

### Mini Nutritional Assessment-Short Form

Nutritional status was assessed using the Mini Nutritional Assessment-Short Form (MNA-SF), which consists of six items with a maximum score of 14 and a minimum score of 0^22^. The MNA-SF scores can also be classified into three groups (normal nutritional status [scores 12–14], at risk of malnutrition [scores 8–11], and malnourished [scores 0–7]).

#### Matsuda–Kanno classification

The Matsuda–Kanno classification was used as a reference for classifying postoperative oral dysfunctions. The classification can be divided into three types: transport, Oral hygiene, and Occlusal. In this study, the cut-off values of each oral function measurement were used as references (Table 1)^11^.

**Table 1:** Matsuda–Kanno classification of postoperative oral dysfunction and cut-off values for oral function measurements.

Types	Names	Definition	Reference values for diagnostic criteria
I	Transport type	A condition in which dysfunction occurs during the oral preparatory and transit phases of swallowing due to treatment-induced damage to the tongue, palate, buccal mucosa, or oral floor	Masticatory function (cut-off value: 83 mg/dL) EAT-10 (cut-off value: 12) Tongue pressure (cut-off value: 14 kPa)
II	Oral hygiene type	A condition in which the self-cleaning and antibacterial moisturizing functions of the oral cavity are impaired by treatment	Number of microorganisms (cut-off value: 10^6.5^ or more) Oral dryness (cut-off value: 27.0) Chief complaint of subjective oral health perception
III	Occlusion type	A condition in which occlusion is impaired due to loss of maxilla and mandible or teeth from treatment	Occlusal force (cut-off value: 230 N)

*EAT-10* eating assessment tool-10.

### Statistical analysis

Descriptive analyses were used to calculate the median, interquartile range (IQR), and relative frequency. For group comparisons, we used the chi-square test and Mann–Whitney U test with Bonferroni correction after the Kruskal–Wallis test as a multiple comparison method. In addition, a trend test (Jonckheere–Terpstra test) was performed. Finally, a multiple regression analysis was conducted with the total MNA-SF score as the objective variable. Statistical analyses were performed using a statistical software (SPSS, Version 26.0; IBM, Armonk, NY, USA). We calculated two-tailed p-values for all analyses, and the alpha level of significance was set at p < 0.05.

## Results

### Patient characteristics

Patient characteristics are summarized in Table 2. This survey included 75 patients who were treated for oral cancer, 52 (69.3%) of whom were male and 23 (30.7%) were female. The median age of the patients was 72.0 years (IQR: 64.0–78.0 years). In addition, 59 (78.7%) patients had a performance status of 0. The tongue was the most frequent primary tumor site, and 31 (41.3%) patients had early stage cancer. Surgery alone was the most common treatment (40 patients, 53.3%). Neck dissection and reconstruction were performed in 48 (64.0%) and 47 (62.7%) patients, respectively. The median number of teeth was 16.0 (IQR: 3.0–24.0). The median (IQR) values of oral function measurements were 3.0 (2.0–5.0), 24.8 (21.3–26.7), 245.6 (18.0–443.6), 17.1 (7.5–23.6), 75.0 (15.0–150.0), and 15.0 (4.0–25.0) for microorganisms (grade), oral dryness, occlusal force (N), tongue pressure (kPa), masticatory function (mg/dL), and EAT-10, respectively. The median FOIS score was 5.0 (IQR: 5.0–6.0), and the median MNA-SF score (malnourished) was 28 (37.3%).

**Table 2:** Demographic and clinical characteristics (N = 75).

Variables	Categories	N (%), median (25–75 percentile)
Sex	Male	52 (69.3)
	Female	23 (30.7)
Age (years)		72.0 (64.0–78.0)
Body mass index (kg/m^2^)		20.4 (18.6–23.6)
Brinkman index		0.0 (0.0–440.0)
Alcohol consumption	Regular drinker	34 (45.3)
	Social drinker	7 (9.3)
	None	34 (45.3)
Number of teeth		16.0 (3.0–24.0)
Systemic disease (yes)	Diabetes mellitus	17 (22.7)
	Hypertension	27 (36.0)
	Cardiovascular disease	8 (10.7)
	Cerebrovascular disease	5 (6.7)
	Liver disease	4 (5.3)
	Pulmonary disease	8 (10.7)
	Kidney disease	5 (6.7)
	Orthopedic disease	8 (10.7)
	Psychiatric disease	6 (8.0)
	Cancer (except oral cancer)	6 (8.0)
Performance status	0	59 (78.7)
	1	9 (12.0)
	2	1 (1.3)
	3	6 (8.0)
Primary tumor site	Tongue	31 (41.3)
	Upper gingiva	16 (21.3)
	Lower gingiva	14 (18.7)
	Palate	3 (4.0)
	Oral floor	5 (6.7)
	Buccal mucosa	3 (4.0)
	Intraosseous of mandible	2 (2.7)
	Lip	1 (1.3)
Tumor stage	Stage I	17 (22.7)
	Stage II	9 (12.0)
	Stage III	12 (16.0)
	Stage IV	37 (49.3)
Treatment	Surgery	40 (53.3)
	Surgery + radiotherapy	10 (13.3)
	Surgery + chemoradiotherapy	25 (33.3)
Neck dissection (yes)		48 (64.0)
Reconstruction (yes)		47 (62.7)
Oral function mesurement	Microorganisms (grade)	3.0 (2.0–5.0)
	Oral dryness	24.8 (21.3–26.7)
	Occulusal force (N)	245.6 (18.0–443.6)
	Tongue pressure (kPa)	17.1 (7.5–23.6)
	Masticatory function (mg/dL)	75.0 (15.0–150.0)
	EAT-10	15.0 (4.0–25.0)
Functional oral intake scale	1	6 (8.0)
	2	4 (5.3)
	3	0 (0.0)
	4	8 (10.7)
	5	22 (29.3)
	6	25 (33.3)
	7	10 (13.3)
MNA-SF	Total score	9.0 (7.0–11.0)
	Normal nutritional status	16 (21.3)
	At risk of malnutrition	31 (41.3)
	Malnourished	28 (37.3)

*MNA-SF* mini nutritional assessment-short form.

### Multiple group comparisons of MNA-SF scores and related factors

Multiple group comparisons of MNA-SF scores and related factors are summarized in Table 3. There were significant differences in age (p = 0.025), body mass index (p = 0.001), number of teeth (p = 0.020), performance status (p = 0.005), tumor stage (p < 0.042) and treatment methods (surgery/surgery and adjuvant radiotherapy/surgery and adjuvant chemoradiotherapy; p < 0.001).

**Table 3:** Group comparisons of MNA-SF and related factors (N = 75).

Variables	Categories	MNA-SF (N [%], mean [SD], or median [25–75 percentile])			p-values
		Normal nutritional status	At risk of malnutrition	Malnourished	
Sex	Male	14 (18.7)	20 (26.7)	18 (24.0)	0.151
	Female	2 (2.7)	11 (14.7)	10 (13.3)	
Age (years)		70 (63.0–73.0)	71.0 (64.0–74.0)	77.5 (71.0–86.0)	0.025*
Body mass index (kg/m^2^)		23.5 (20.2–24.8)	21.1 (19.3–23.7)	19.0 (17.0–86.0)	0.001*
Brinkman index		200.0 (0.0–440.0)	0.0 (0.0–400.0)	0.0 (0.0–525.0)	0.313
Number of teeth		21.0 (10.5–26.0)	20.0 (8.0–26.0)	6.5 (0.0–21.5)	0.020*
Performance status		0.0 (0.0–0.0)	0.0 (0.0–0.0)	0.0 (0.0–1.0)	0.005*
Primary tumor site	Tongue	6 (8.0)	13 (17.3)	12 (16.0)	0.937
	Gingiva	5 (6.7)	14 (18.7)	11 (14.7)	0.647
	Others	5 (6.7)	4 (5.3)	5 (6.7)	0.331
Tumor stage		3.0 (1.0–4.0)	3.0 (1.0–4.0)	4.0 (3.0–4.0)	0.042*
Treatment	Surgery	12 (16.0)	21 (28.0)	7 (9.3)	0.001*
	Surgery + Radiotherapy	1 (1.3)	1 (1.3)	8 (10.7)	
	Surgery + Chemoradiotherapy	3 (4.0)	9 (12.0)	13 (17.3)	
Neck dissection (yes)		10 (13.3)	17 (22.7)	21 (28.0)	0.263
Reconstruction (yes)		9 (12.0)	18 (24.0)	20 (26.7)	0.471

*MNA-SF* mini nutritional assessment-short form, *SD* standard deviation.

*p < 0.05.

### Multiple group comparisons of MNA-SF scores and oral function measurements

**Figure 2:**
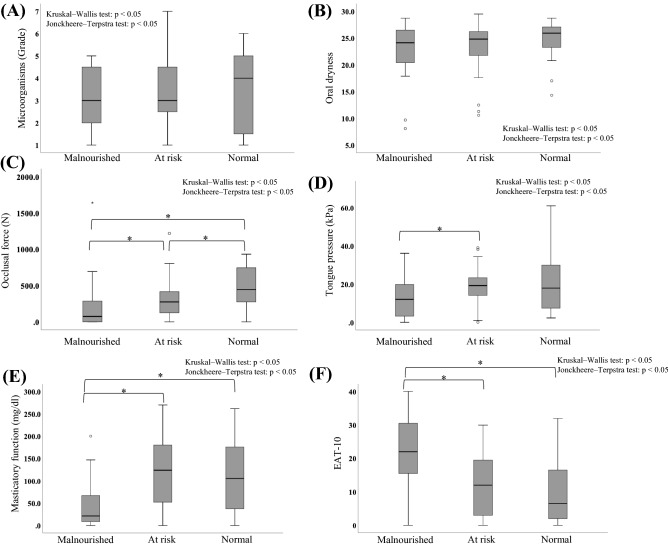
Multiple group comparisons of MNA-SF scores and oral function measurements. (**A**) microorganisms; (**B**) oral dryness; (**C**) occlusal force; (**D**) tongue pressure; (**E**) masticatory function; (**F**) EAT-10; *MNA-SF* mini nutritional assessment-short form, *EAT-10* eating assessment tool-10.

Multiple group comparisons of MNA-SF scores and oral function measurements are shown in Fig. 2. There were significant differences in occlusal force, tongue pressure, masticatory function, and EAT-10 levels.

### Relationship between MNA-SF and oral function measurement using multiple regression analysis

Multiple regression analysis showed a statistically significant association with MNA-SF in terms of masticatory function (β = 0.28, p = 0.012) and EAT-10 levels (β = − 0.32, p = 0.007; Table 4).

**Table 4:** Relationship between MNA-SF and oral function measurement using multiple regression analysis.

Variables	β	B	95% confidence interval		p-value	Adjusted R^2^
			Lower	Upper		
Microorganisms (grade)	0.06	0.12	− 0.34	0.58	0.602	0.27
Oral dryness	0.13	0.09	− 0.06	0.23	0.233	0.28
Occulusal force (N)	0.21	< 0.01	< 0.01	< 0.01	0.066	0.30
Tongue pressure (kPa)	0.19	0.05	− 0.01	0.11	0.093	0.29
Masticatory function (mg/dL)	0.28	0.01	0.003	0.02	0.012*	0.33
EAT-10	− 0.32	− 0.09	− 0.15	− 0.03	0.007*	0.34

In the multiple regression analysis, analyses were separated for each oral function, and sex, age, tumor stage, treatment, and primary tumor site were simultaneously forced into the model equation for each analysis to adjust for confounding factors.

*MNA-SF* mini nutritional assessment-short form, *β* standardized partial regression coefficient, *B* partial regression coefficient.

*p < 0.05.

## Discussion

The major finding of this study is that multiple oral dysfunctions have an impact on nutritional status. Of these, masticatory function and EAT-10 levels were found to be independent and distinct oral dysfunctions in our previous studies^11^. In addition, the EAT-10 swallowing assessment has traditionally been associated with nutritional status in healthy individuals^23^. This disproved our null hypothesis and the alternative hypothesis was adopted. Our hypothesis that “different types of postoperative oral dysfunction require different nutritional guidance” is likely to be correct, and our results were reasonable. In other words, the Type I (Transport type) and III (Occlusion type) hypotheses were accepted, but the Type II (Oral hygiene type) oral dysfunction hypothesis was rejected.

Masticatory function and EAT-10 constitute Type I. Arthur et al. reported that there was no relationship between masticatory function and nutritional status, but this may be due to the fact that the assessment items were the number of teeth and occluding pairs of teeth, rather than masticatory function, which is the ability to pull back food from the anterior region of the mouth to the molar region and squeeze back processed food from the mouth to the pharynx^24^. Therefore, based on the results of our study, we believe that masticatory function is related to postoperative nutritional status. Since it is well known that tongue pressure and masticatory function decrease with resection, especially in patients with oral cancer, our results also support the results of previous studies^25,26^. In addition, a cross-sectional study of 909 hospitalized patients showed an association between EAT-10 levels and nutritional status, and the results of this study were similar^27^. Therefore, malnutrition may occur due to Type I. In the nutritional instructions for Type I, it is especially important to choose a food texture that facilitates the formation of boluses, which can be transported to the pharynx by gravity^28^. In addition, the use of palatal augmentation prosthesis as a patient specific oral-maxillofacial prosthetic treatment makes it easier to direct the bolus into the esophagus, and rehabilitation with maneuvers on swallowing function is a reinstatement of safe oral intake^29,30^.

In contrast, multivariate analysis did not show a significant association between occlusal force and nutritional status, but occlusal force was the only component of Type III, suggesting the possibility of nutritional impairment caused by Type III. Decreased occlusal force is mainly caused by resection of the masticatory muscles (temporalis, masseter, lateral pterygoid, and medial pterygoid muscles) and loss of occluding pairs of teeth due to maxillary or mandibular resection^31^. This study is the first to show that maxillary and mandibular deficiencies can reduce occlusal force and affect nutritional status, while also suggesting that oral-maxillofacial prosthetic treatment may be useful. The first-line treatment for patients with maxillary or mandibular defects is patient-specific oral-maxillofacial prosthetic treatment using dentures and dental implants^32^. However, prosthetic treatment has some limitations, and even with dental implants, it is unlikely that the occlusal force will be restored to what it was before resection^33^. Decreased occlusal force is mainly associated with decreased intake of vegetables and proteins, suggesting that nutritional guidance should pay more attention to the loss of food diversity than food texture^34^. In addition, since regular and longitudinal dental visits are important for oral-maxillofacial prosthetic management and care, community collaboration is also important from the perspective of long-term nutritional management following oral cancer treatment^35^.

The results of the trend test suggested that malnutrition caused by the Type I and Type III described above may be exacerbated in stages. In other words, in clinical practice, we should not only focus on the presence of malnutrition, but also screen and intervene earlier for patients in the pre-malnutrition or intermediate stages of malnutrition using a multidisciplinary team approach^36^.

We can consider several limitations to our study, the main ones being its retrospective nature and small sample size due to the study period and its single-center design^37^. Given the limited number of items that could be classified as explanatory variables, only rough adjustment for confounders was conducted for tumor site and treatment. In addition, sub-group analysis was not conducted. However, since postoperative oral dysfunction and dysphagia have been reported to occur in both single and multimodal treatments, it was assumed that an analysis considering more confounding factors would yield results similar to those of this study^38^. Further, because many patients in this study had advanced oral cancer, the prevalence of malnutrition due to postoperative oral dysfunction may need to be estimated lower when considering generalizability^39^. However, since the number of severely ill patients is expected to increase in Japan, where the population is super-aging, the generalizability of this study is likely to be high in developed countries with aging populations^40^. Future studies should evaluate whether malnutrition is reversible over time because the preoperative oral function and the long-term prognosis for postoperative oral function are unclear.

## Conclusion

Decreased masticatory function and EAT-10 levels are risk factors for malnutrition. Postoperative oral dysfunction Type I (Transport type) may be a risk factor for nutritional status in patients treated for oral cancer. Further, individual nutritional guidance may be adapted to each type of postoperative oral dysfunction.

## Data Availability

The datasets used and/or analysed during the current study available from the corresponding author on reasonable request.
